# From memory clinics to Brain Health Services: implications for older adults and the future of cognitive care in Europe

**DOI:** 10.1007/s41999-026-01557-0

**Published:** 2026-08-05

**Authors:** T. J. Welsh, Y. Chen, S. P. Kennelly, T. J. Quinn, D. Religa, S. D. Shenkin, P. Soysal, H. M. Roitto

**Affiliations:** 1 Research Institute for Brain Health, ReMindUK, Bath, UK; 2 Royal United Hospitals Bath NHS Foundation Trust, Bath, UK; 3 Bristol Medical School, University of Bristol, Bristol, UK; 4 Univ. Lille, Lnserm, CHU Lille, JUNIA, U1352 - BioPrev - Bien vieillir: de l’inflammaging à la prévention, 59000 Lille, France; 5 Institute of Memory and Cognition, Tallaght University Hospital, Dublin, Ireland; 6 Department of Medical Gerontology, School of Medicine, Trinity College Dublin, Dublin, Ireland; 7 School of Cardiovascular and Metabolic Health, University of Glasgow, Glasgow, UK; 8 Scottish Funding Council Brain Health ARC, Glasgow, UK; 9 Department of Neurobiology, Care Sciences and Society, Karolinska Institutet, Stockholm, Sweden; 10 Ageing and Health, Usher Institute, University of Edinburgh, Edinburgh, UK; 11 Department of Geriatric Medicine, Faculty of Medicine, Bezmialem Vakif University, Adnan Menderes Bulvarı (Vatan Street), 34093 Istanbul, Fatih Türkiye; 12 Faculty of Medicine and Health Technology, Department of Geriatrics, Tampere University, Tampere University Hospital, Wellbeing Services County of Pirkanmaa, Tampere, Finland

**Keywords:** Brain Health Services (BHS), Older adults, Dementia prevention, Comprehensive Geriatric Assessment (CGA), Equity of access

## Abstract

**Aim:**

To explore the implications of transitioning from traditional memory clinics to Brain Health Services (BHS) for older adults, and to advocate for a geriatric-informed, equitable, and function-oriented approach to brain health care.

**Methods:**

This perspective article draws on discussions within the EuGMS Brain Health and Dementia Specialist Interest Group and relevant literature to examine the implications of emerging Brain Health Services for older adults.

**Results:**

The emergence of Brain Health Services represents a significant shift in cognitive healthcare, moving beyond traditional memory clinic models focussed primarily on dementia diagnosis and management towards prevention, risk reduction, and maintenance of cognitive function. Potential benefits include earlier engagement with cognitive care, multidomain lifestyle interventions, reduction of stigma surrounding dementia, and facilitation of access to emerging disease-modifying therapies. However, substantial challenges remain. Biomarker-driven approaches may overemphasise Alzheimer’s disease-centric frameworks despite the high prevalence of mixed pathologies and complex comorbidity in older adults. Ethical concerns surrounding risk disclosure, inequitable access, and resource allocation are also considerable, particularly if services disproportionately benefit younger, healthier, or socioeconomically advantaged populations.

**Conclusions:**

Brain Health Services should be reframed through a gerontologically informed lens that prioritises function, independence, quality of life, and equitable access alongside prevention. Integration with comprehensive geriatric assessment and existing older persons’ care pathways is essential. Future research should focus on pragmatic real-world evaluation, inclusive outcome measures, and personalised approaches that account for heterogeneity in ageing. Ultimately, the success of BHS will depend on their ability to support healthy ageing while remaining responsive to the lived realities and priorities of older adults.

## Key summary points

**Aim:**

To explore the implications of transitioning from traditional memory clinics to Brain Health Services (BHS) for older adults, and to advocate for a geriatric-informed, equitable, and function-oriented approach to brain health care.

**Findings:**

BHS offer opportunities for earlier engagement, multidomain risk reduction, and integration of emerging disease-modifying therapies. However, current models risk overemphasising biomarkers and prevention while overlooking frailty, multimorbidity, functional outcomes, and inequities affecting older adults. Existing evidence for effectiveness and cost-effectiveness remains limited, particularly in real-world older populations.

**Message:**

Brain Health Services should complement rather than replace existing cognitive and geriatric care pathways. Future BHS models must prioritise holistic, person-centred care, equity of access, functional independence, and integration within comprehensive geriatric assessment frameworks.

**Graphical abstract:**

## Graphical abstract

**Figure:**
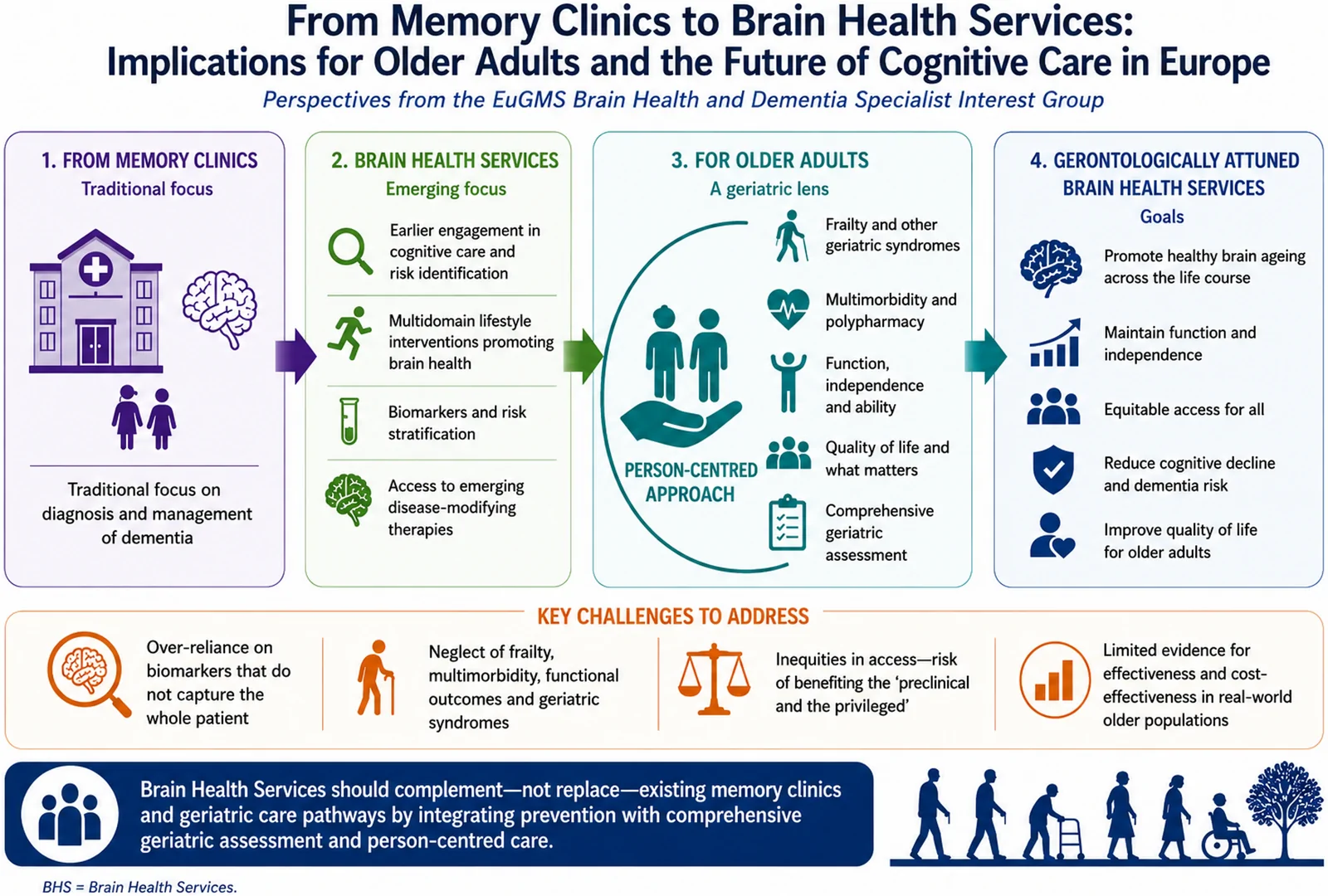

## Introduction

The shifting landscape of cognitive health services marks a pivotal moment in the care of older people. With rising life expectancy and the growing prevalence of neurodegenerative diseases such as Alzheimer’s disease (AD) and other causes of dementia, the traditional role of memory clinics is being re-evaluated. A different model, Brain Health Services (BHS), has emerged, placing greater emphasis on prevention, risk reduction, and enhancement of cognitive function, rather than only diagnosis and disease management [1].

This article considers the implications for older adults, especially frail older adults, of a shift to BHS. While the concept is appealing, and potentially transformative, the practical and ethical complexities are considerable. There is a risk that BHS evolve into services for the ‘preclinical and the privileged’, rather than those at greatest clinical need. This perspective piece argues that while BHS represent an exciting evolution in neurocognitive care, their current conceptualisation risks misalignment with the realities of ageing, and risks marginalising the most vulnerable in our society.

We propose that BHS must be reframed through a gerontologically attuned lens, which explicitly incorporates frailty, other geriatric syndromes, multimorbidity, competing health priorities, and equity of access, and considers the diagnosis and management of cognitive impairment and dementia, as well as championing interventions that may reduce the risk of developing these conditions. Without this, there is a risk that BHS will preferentially benefit younger, healthier populations while failing to address the needs of older adults who carry the greatest burden of cognitive and associated functional impairment. We therefore advocate for a model of geriatric-informed, equitable BHS, integrated within existing older care pathways rather than developed in parallel.

### Development of this perspective

This article reflects discussions within the EuGMS Brain Health and Dementia Specialist Interest Group. We have held in-person and online meetings where multidisciplinary international interest holders discussed the current state, and future, of Brain Health Services for older adults. This piece represents a synthesis of these discussions additionally informed by scoping reviews of relevant literature [2, 3].

### Clinical context

Traditionally, memory clinics have focussed on the identification, staging, and management of dementia. This model serves a clear purpose: an accurate and timely dementia diagnosis offering diagnostic clarity, access to post-diagnostic support, and facilitating access to broader services. However, in many real-world settings, a significant proportion of memory clinic attendees present with mild cognitive impairment (MCI), subjective cognitive concerns (Subjective cognitive impairment SCI), or non-neurodegenerative causes of cognitive decline. This highlights the need for a broader, more inclusive model of care.

In contrast, BHS advocate a preventive, proactive framework targeting those with subjective cognitive decline, MCI, and asymptomatic individuals deemed ‘at risk’ [4]. It recognises that brain health exists along a continuum rather than a binary disease/no disease model. It uses structured risk communication and multidomain interventions targeting vascular health, lifestyle modification, cognitive training, and sometimes neuromodulation techniques [5].

Biomarker-driven approaches, e.g. APOE genotyping, cerebrospinal fluid (CSF) and plasma biomarkers, and neuroimaging (e.g. amyloid PET), are often emphasised within emerging BHS models and AD-focussed frameworks [1]; these technologies are not universally available and may not be appropriate or necessary for all individuals or healthcare systems. Over-reliance on such approaches risks narrowing the scope of BHS and reinforcing an AD-centric model.

In older adults, coexisting pathologies are common. AD and cerebrovascular disease frequently coexist, and geriatric syndromes including frailty, polypharmacy, malnutrition, sleep disturbances, depression and falls are highly prevalent [6]. BHS therefore present an opportunity to build on the strengths of geriatric medicine: holistic, person-centred, and function-orientated care. Rather than focussing on only cognitive reserve, services should assess and support overall functional ability, independence, and quality of life. These distinctions suggest that a direct translation of BHS models developed in younger or selected populations may not be appropriate for older adults without significant adaptation (see Table 1).

**Table 1:** Key differences in Brain Health Service application across the life course

Domain	Midlife/younger populations	Older adults
Risk horizon	Long-term prevention (10–30 years)	Shorter, uncertain time-to-benefit
Pathology	Often single-disease focus (AD, vascular, Lewy body, etc.)	Mixed pathologies (AD, vascular, Lewy body, etc.)
Biomarkers	Higher predictive utility	Reduced specificity; discordance common
Intervention goals	Prevention of decline	Prevention of decline, maintenance of function, independence, and quality of life
Comorbidity	Lower burden	High multimorbidity, polypharmacy, frailty
Patient priorities	Risk reduction	Symptom burden, function, caregiver support
Health system role	Preventive	Preventive + supportive + compensatory + advanced care planning

### Clinical implications of a shift to Brain Health Services

#### Benefits

1. Reframing care towards risk reduction and function
  Brain Health Services reflect an evolving approach to cognitive disorders, moving beyond diagnosis towards risk reduction and support for functional ability. Rather than waiting for decline, these services provide a model where individuals actively engage in preserving their cognitive capital. This aligns with broader gerontological principles of intrinsic capacity, healthy ageing, and resilience.
2. Potential for multidomain intervention benefit
  The multidomain intervention approach, exemplified by trials such as FINGER [5], MAPT [6], preDIVA [7], and POINTER [8], shows some promise. Although findings are mixed, these studies support the idea that physical activity, social engagement, cognitive training, and vascular risk factor control may have modest benefits for cognitive as well as physical and functional outcomes. For selected older individuals, particularly those with modifiable cardiovascular risk, BHS could deliver tangible improvements in quality of life and function.
3. Reduction in stigma and earlier engagement
  Importantly, this approach may also help reduce stigma. By positioning brain health as part of routine well-being—akin to heart or bone health—BHS could normalise help-seeking and facilitate earlier engagement with services. This could be particularly helpful in communities where dementia remains stigmatised or misunderstood.
4. Gateway to emerging therapies
  BHS may facilitate a more timely diagnosis of dementia or other clinical conditions, which is increasingly relevant in the context of emerging disease-modifying therapies (DMTs) for AD [8]. Importantly, BHS could function not only as preventive services but also as gateways to DMTs. This dual role introduces both opportunity and tension. While early identification may facilitate access to emerging treatments, it also risks reorienting BHS towards biomarker-driven selection pathways that prioritise treatment eligibility over holistic brain and physical health.

### Challenges and unintended consequences of a shift to Brain Health Services

1. Evidence gaps and uncertain effectiveness
  While the concept of risk reduction is compelling, the evidence base underpinning BHS is still evolving. Biomarker-based risk stratification in older adults remains imperfect, particularly in the “oldest old” or those with multiple comorbidities [9]. The focus on biomarkers risks the over-simplification of brain health into a binary disease/no disease model. The role of the geriatrician here is clear—to advocate for a person-centred, holistic assessment—particularly in the oldest old or those living with frailty [10]. Many proposed interventions have only modest effect sizes, and their cost-effectiveness is unclear. Without robust, long-term trial data demonstrating reduced incidence of dementia or improved outcomes, the risk of overpromising benefit remains high, and there is a significant risk that individuals who go on to be diagnosed with dementia will feel that they have ‘failed’ in preventing this due to their own actions.
2. Resource allocation and service overlap
  The integration of BHS into existing healthcare systems raises logistical and financial concerns. Should health systems fund biomarkers, imaging and cognitive interventions for people with SCD, when those with established dementia still face unmet needs? [11] There is a real danger that limited resources will be diverted away from those with functional impairment or socioeconomic vulnerability. Services may become disproportionately focussed on identifying individuals suitable for DMTs who are typically younger and less frail, thereby reinforcing inequities in access and diverting attention from broader supportive and preventive interventions that may benefit a wider ageing population. BHS should stress that brain health can be optimised whether someone has a neurodegenerative disease or not.
  Resource considerations extend beyond financial investment. There is a risk that workforce interest and training may preferentially shift towards technologically driven, biomarker-focussed services, potentially drawing attention away from the complex, multidisciplinary care required for frail older adults. Conversely, geriatric medicine may have a central role in delivering equitable and holistic BHS where many components of multidomain interventions closely resemble established principles of Comprehensive Geriatric Assessment (CGA) [12].
3. Ethical complexity of prevention in later life
  Preventive models assume that there is sufficient time to intervene meaningfully. Yet, many individuals attending BHS will already be in mid to later life. The window of modifiability may be narrow or uncertain, but this should not lead us to nihilism; there is always scope for a care plan to optimise brain health [3]. A central ethical challenge lies in the balance between empowerment and burden. On one hand, BHS aim to provide individuals with actionable strategies to optimise brain health. On the other, they risk placing responsibility on individuals to modify risk factors that may be only partially controllable, particularly in the context of ageing, multimorbidity, and socioeconomic constraints [13]. There is a danger that brain health messaging may inadvertently become moralising, framing cognitive decline as a failure of prevention [14] rather than a complex biological and social process.
  The psychological impact of risk disclosure must also be considered [15]. Communicating elevated dementia risk, especially if done in the context of a disproportionate emphasis on biomarker results, may lead to anxiety, stigma, or altered self-perception without clear therapeutic benefit. This is especially relevant in older adults, where competing health priorities may outweigh the perceived value of risk information.
  Finally, there are broader issues of justice and equity. If access to BHS is skewed towards individuals with higher health literacy, digital access, or socioeconomic advantage, these services may widen existing disparities in cognitive health [16]. Ethical implementation therefore requires careful attention to inclusivity, proportionality of intervention, and alignment with patient priorities, especially where older persons are concerned.

## Research implications

The advent of BHS presents fertile ground for research, but priorities must be carefully selected.

First, there is a pressing need for real-world evidence. Trials such as FINGER [17], MAPT [6], preDIVA [7], and POINTER [8] have typically involved selected populations in controlled environments. Pragmatic trials, embedded in routine care, and including older adults with comorbidities and polypharmacy, are needed to assess the true utility of BHS.

Second, the development of accessible biomarkers—particularly blood-based assays—could democratise BHS by removing reliance on costly or invasive procedures. However, their predictive validity, including in older diverse populations, must be critically appraised [18] and biomarkers alone are not sufficient—a clinical-biological approach is needed.

Third, personalised approaches require far greater understanding of heterogeneity in ageing. The interaction of genetic, lifestyle, and psychosocial factors across the life course must be unpacked. Frailty, multimorbidity, inflammaging, and neurovascular resilience are likely to moderate intervention responses and must be embedded in future models.

Lastly, outcome measures in BHS research must reflect what matters to older adults. So far, there is no consensus for outcome measures in BHS, even for younger adults. Delayed conversion to dementia is important, but so too are preservation of daily function, emotional well-being, and autonomy. These broader outcomes must be included in research design.

## Educational and professional development needs

The shift to BHS requires a recalibration of professional education.

Delivering BHS at scale requires a workforce trained not only in cognitive assessment, but also in genomics, shared decision-making, risk counselling and behavioural change techniques. This represents a significant educational challenge for psychiatry, geriatrics, primary care, and neurology [19]. Without adequate training, there is a risk that clinicians may miscommunicate risk, or fail to address psychological harms arising from ambiguous results.

For the public, education must go beyond lifestyle advice. It must confront the complexity of brain health—acknowledging that while some risk factors, e.g. vascular, are modifiable, age and genetics are not. Public messaging must avoid blame narratives or oversimplified slogans (“prevention” is unlikely, whereas “modification” or “risk reduction” is possible). The relative impacts of DMTs and lifestyle factors should be communicated.

Furthermore, peer support, group interventions and social prescribing may have a role in advancing BHS beyond the clinic setting. BHS programmes, such as those delivered in Switzerland [20], particularly those tailored to ethnically and socioeconomically diverse groups, warrant investment and evaluation.

## Policy and health system considerations

Policymakers face a dilemma [2]. While dementia prevention is a public health priority, the evidence base for current BHS interventions remains inconclusive. Funding decisions must weigh short-term costs against long-term gains and consider both equity and opportunity cost. A key unresolved question is where BHS should sit within healthcare systems. We propose that, rather than functioning as standalone specialist services, BHS should be embedded within integrated care pathways, linking primary care, geriatric medicine, and specialist cognitive services.

Reimbursement structures need consideration and revision. Current systems often favour acute care or pharmacological treatments. For BHS to thrive, preventive services—many of which are behavioural or lifestyle-based—must be valued and incentivised.

There is also a risk that BHS will become disproportionately available to those with higher education, digital literacy [21] and healthcare engagement. Policy must prioritise inclusivity—embedding BHS within primary care, community services, and existing healthy ageing programmes. Guidelines are needed to define core components of BHS, stratify individuals based on likely benefit, and standardise risk communication. Without this, variability in service quality and accessibility will widen health inequalities.

## Conclusion

Brain Health Services represent a bold reimagining of cognitive care for older people. Their preventive ethos, personalised assessments and multidomain interventions offer a welcome alternative to the reactive nature often associated with dementia care. However, their successful implementation in older populations requires careful consideration.

BHS must move beyond a predominantly biomarker-driven and prevention-focussed model to one that is gerontologically attuned, function-orientated, and equity-focussed. In older adults, the goals of care extend beyond delaying disease onset to include maintaining independence, supporting quality of life, and addressing the realities of multimorbidity and frailty. As BHS evolve, they must be critically evaluated, equitably delivered, and implemented in a manner that is sensitive to existing services. Brain health is for everyone, and it remains relevant across the life course—including in older age and after a diagnosis of a neurodegenerative condition. A move to a Brain Health focus through Brain Health Services and wider public health messaging has the potential to promote healthy ageing in its broadest sense.

Brain Health Services must be a tool to support healthy ageing but not at the cost of restricting access to timely and accurate cognitive diagnosis. We therefore argue that the future of BHS lies not in replacing existing services but in integrating brain health into comprehensive geriatric care frameworks. Their value will ultimately depend not on innovation alone, but on their ability to align with the lived realities of ageing and the priorities of older adults.
